# Mapping a novel positive allosteric modulator binding site in the central vestibule region of human P2X7

**DOI:** 10.1038/s41598-019-39771-5

**Published:** 2019-03-01

**Authors:** Stefan M. Bidula, Brett A. Cromer, Samuel Walpole, Jesus Angulo, Leanne Stokes

**Affiliations:** 1 School of Pharmacy, University of East Anglia, Norwich Research Park, Norwich, NR4 7TJ United Kingdom; 2 School of Medical Sciences, RMIT University, Bundoora, VIC 3083 Australia; 3 Present Address: Department of Chemistry & Biotechnology, Swinburne University of Technology, Hawthorn, VIC Australia

## Abstract

P2X7 receptors are important in the regulation of inflammatory responses and immune responses to intracellular pathogens such as *Mycobacterium tuberculosis* and *Toxoplasma gondii*. Enhancement of P2X7 receptor responses may be useful in pathogen clearance particularly in individuals with defective microbial killing mechanisms. Ginsenosides from *Panax ginseng* have been discovered to act as positive allosteric modulators of P2X7. Here we describe a novel modulator binding site identified by computational docking located in the central vestibule of P2X7 involving S60, D318, and L320 in the lower body β-sheets lining the lateral portals. Potentiation of ATP-mediated responses by ginsenosides CK and Rd caused enhanced ionic currents, Ca^2+^ influx and YOPRO-1 uptake in stably transfected HEK-293 cells (HEK-hP2X7) plus enhanced cell death responses. Potentiation of ATP responses by CK and Rd was markedly reduced by mutations S59A, S60A, D318L and L320A supporting the proposed allosteric modulator binding site. Furthermore, mutation of the conserved residues S60 and D318 led to alterations in P2X7 response and a higher sensitivity to ATP in the absence of modulators suggesting residues in the connecting rods play an important role in regulating P2X7 gating. Identification of this novel binding site location in the central vestibule may also be relevant for structurally similar channels.

## Introduction

P2X7 is an ATP-gated ion channel expressed on various immune cell populations including monocytes and macrophages^1^. P2X7 activation results in the activation of the NLRP3-caspase 1 inflammasome^2^, release of pro-inflammatory mediators (e.g. IL-1β, IL-18)^3^, shedding of transmembrane proteins (e.g. CD62L, CD23)^4^, and occurrence of cell death^5^. Moreover, P2X7 is implicated in immunity to intracellular pathogens such as *Mycobacterium tuberculosis* and *Toxoplasma gondii* amongst others making it a potential target for the modulation of the immune response^6,7^.

Ginseng has been utilised throughout Asia for centuries where it is considered a ‘panacea’, promoting longevity and treating many diseases, although the exact mechanisms by which ginseng can exert these biological effects are not fully understood^8,9^. The ginseng root extract contains a complex mixture of bioactive compounds such as polysaccharides, glycolipoproteins, and polyacetylenic alcohols, however the most extensively studied compounds are the ‘steroid-like’ dammarane triterpenoid glycosides termed ginsenosides^8^. Ginsenosides can be further divided into the protopanaxadiols (e.g. Rb1, Rb2, Rc, Rd, Rg3, Rh2), and the protopanaxatriols (e.g. Re, Rf, Rg1, Rg2, Rh1), based upon whether sugar moieties are attached to carbon-3 (diols) or carbon-6 (triols). Metabolism of ginsenosides in the gut by microbial flora species results in the formation of readily absorbable metabolites including the ginsenoside compound K (CK)^10^. Ginsenosides have been demonstrated to modulate a plethora of immune cell functions such as phagocytosis, the function of inflammatory enzymes, the production of both pro- and anti-inflammatory cytokines, the modulation of intracellular signalling pathways, and regulation of the inflammasome^9^. Moreover, there is increasing evidence of the potential antimicrobial properties of ginseng, with ginsenosides providing protection against viruses (HIV, influenza, respiratory syncytial virus), bacteria (*E. coli*, *Staphylococcus aureus, Brucella abortus, Pseudomonas aeruginosa*) and fungi (*Candida albicans*)^9,11,12^.

We recently identified that the activity of P2X7 receptors could be modulated by protopanaxadiol ginsenosides such as CK, Rd, Rb1 and Rh2^13^. These chemicals could enhance the action of the physiological agonist ATP, increasing both the sensitivity and the maximum response of P2X7 to agonist, thus defining the ginsenosides as positive allosteric modulators^13^. To date, there have been few positive allosteric modulators (PAMs) identified for P2X7 including clemastine, polymyxin B, ivermectin and tenidap^14–16^ and currently nothing is known about their molecular binding sites. Much more is known about negative allosteric modulator binding to P2X7 with a detailed allosteric binding site recently being described for AZ10606120 that may also accommodate other antagonists including JNJ-47965567, A804598, and A740003^17,18^. Most of what we know about P2X receptor structure comes from the crystal structures of closed and open state zebrafish P2X4 (zfP2X4), giant panda P2X7 (pdP2X7) and human P2X3 receptors^17,19^ plus detailed mutagenesis studies investigating ectodomain and transmembrane domain movements^18,20–22^. Our aim in this study was to identify the putative molecular binding site for ginsenoside PAMs on the P2X7 receptor using a molecular modelling approach combined with site-directed mutagenesis. We describe a novel modulator binding site located in the central vestibule region of P2X7 involving two β-sheets in the lower body region enclosing this cavity. We demonstrate that mutations of two key residues, D318 and L320, abolished potentiation of P2X7 responses by the ginsenosides CK and Rd.

## Results

### Identification of a putative ginsenoside binding pocket by molecular modelling

**Figure 1:**
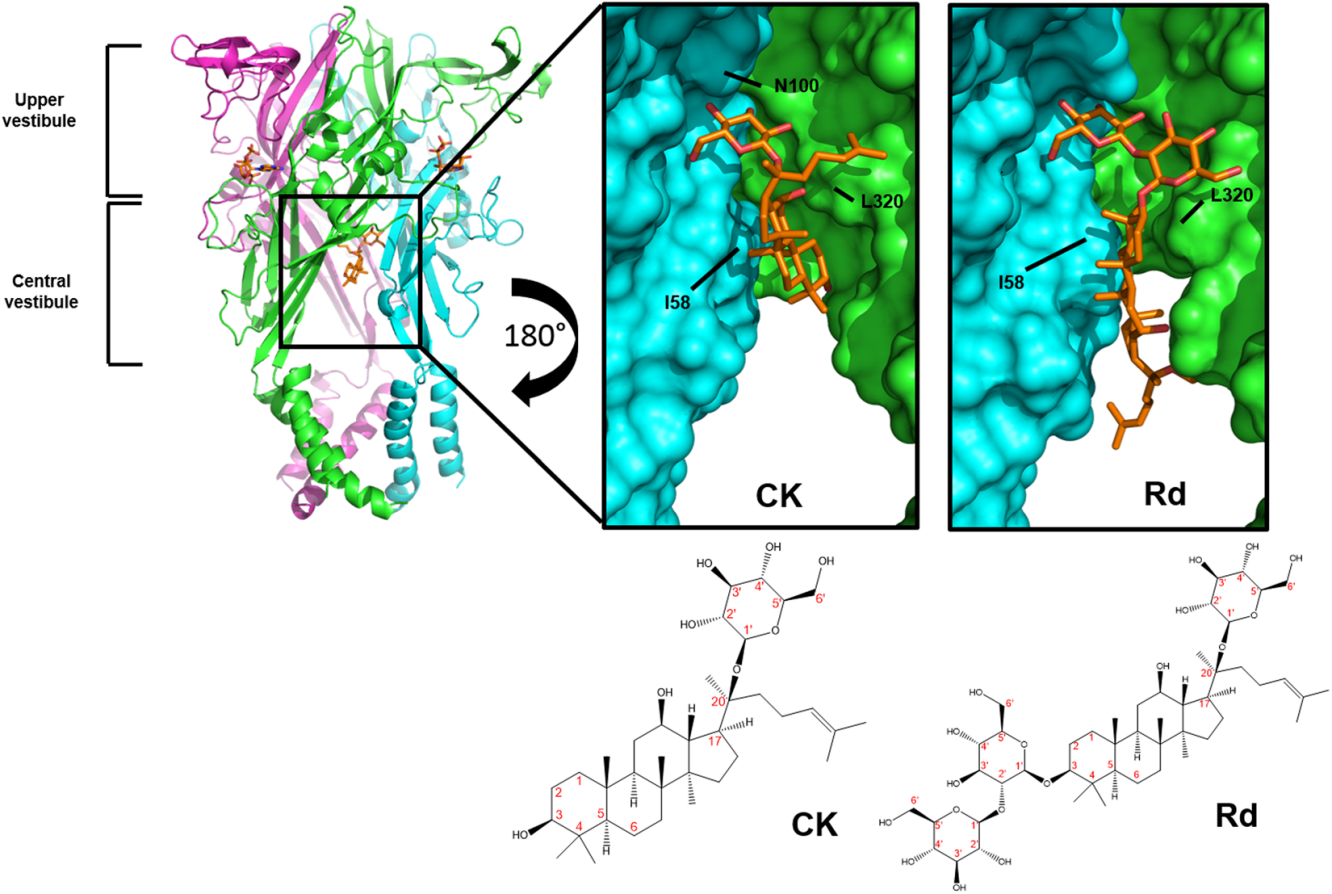
Ginsenosides bind to the central vestibular region of P2X7. Representation of a homology model of hP2X7 trimer in the open state with ginsenoside CK docked in the central vestibular region. Each subunit is differentially coloured (green, cyan, magenta). Panels show a zoom-in and rotated view of the binding site from the inside of the cavity facing outwards (rotation of 180°). Surface rendered images are shown to visualise the binding pocket for CK and Rd on hP2X7. Ligands and side chains are represented as sticks with all hydrogen atoms omitted for clarity. Two-dimensional chemical structures of CK and Rd are also displayed with carbon atoms labelled in red.

**Figure 2:**
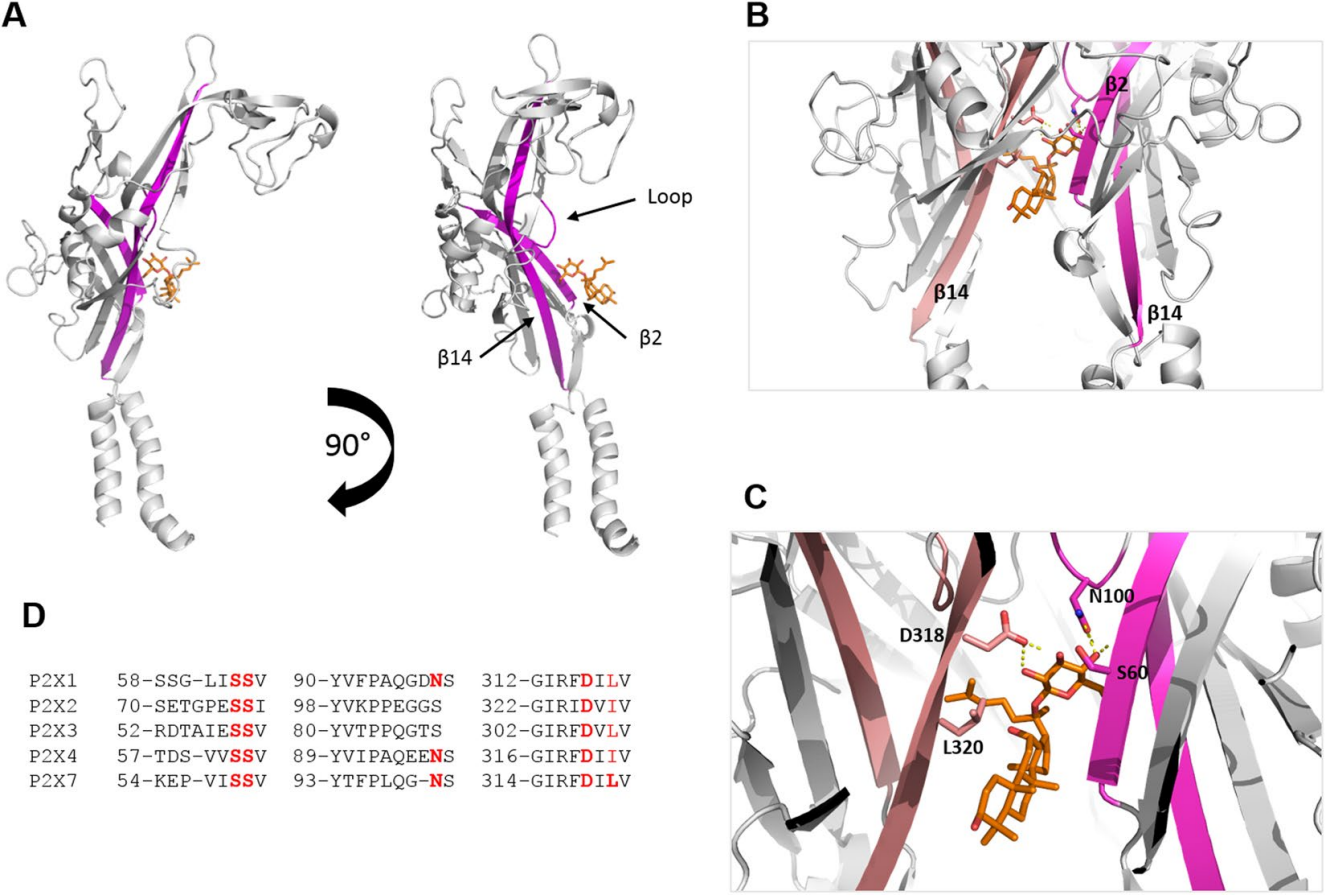
Ginsenoside binding pocket consists of interactions with D318, N100 and S60 residues in hP2X7 lower body region. (**A**) An individual subunit is displayed with two lower body region β-sheets highlighted in magenta (β2 and β14). CK is shown docked into the putative binding site. Rotation of this individual subunit by 90° allows better visualisation of the internal loop containing N100 that is predicted to make contact with the glucopyranose ring of ginsenoside CK. (**B**) Trimeric view of the hP2X7 model showing the docked ginsenoside connecting inter-subunit β-sheets β2 (magenta) and β14 (salmon pink). (**C**) Closer view of the docked ginsenoside CK displaying interacting residues predicted to make polar contacts; D318, N100 and S60.

We previously demonstrated that ginsenoside Rd and the major *in vivo* metabolite CK, act as positive allosteric modulators of human P2X7 receptors requiring the presence of agonist (ATP) for their action. This, together with data suggesting pre-treatment with P2X7 antagonists such as AZ10606120 could prevent P2X7 opening even in the presence of CK^13^ led to the hypothesis that ginsenosides may bind to the open ATP-bound conformation of P2X7. To investigate this we generated homology models of human P2X7 (hP2X7) based upon the closed and open states of zfP2X4 (pdb 3H9V and 4DW1)^19,23^ and the closed state of pdP2X7 (pdb 5U1L)^17^. Model building and preliminary docking of the ginsenosides CK and Rd to human P2X7 was carried out using Swissmodel and Autodock Vina respectively, as previously described for P2X4^24^. Subsequent analysis to further define the binding pocket was performed using consecutive runs of Sitemap software and docking calculations with Glide. The best predicted binding site for ginsenosides is located within the central vestibule of hP2X7 in a large hydrophobic cleft lined by β-sheets that define the lower body wall of the trimeric channel (Fig. 1). This novel binding pocket could be defined as being inter-subunit since interactions are made with residues in the β2-sheet on one subunit and residues in the β-14 sheet on the adjacent subunit (Fig. 2A,B)) thus connecting the post-TM1 region on one subunit with the pre-TM2 region on the adjacent subunit. Three internal loops at the top of the central vestibule (one from each subunit) guard the upper boundary of this lower body cavity (Fig. 2A,B) and connect the lower and upper body regions.

Induced fit docking calculations using Glide revealed two elements to the putative ginsenoside binding site; a glucose-specific subsite located close to the top of the central vestibule cavity and a hydrophobic cleft co-ordinating the steroid backbone of the ginsenosides (Figs 1 and 2C). All protopanaxadiol ginsenosides with activity at P2X7 could be docked into the same binding pocket on the open hP2X7 model (Fig. S1).

We focused on elucidating the binding of CK and Rd in detail as these have the highest positive modulator activity at hP2X7^13^. These two ginsenosides have highly similar structures with a single glucopyranose ring attached to carbon-20 (Fig. 1) although Rd has an additional disaccharide moiety attached to carbon-3. The binding of the glucopyranose ring of CK to hP2X7 makes hydrogen bond interactions with residues S60, N100 and D318 (Fig. 2C). In particular it is the hydroxyl groups on carbons 2′ and 3′ of the glucopyranose moiety that interact with the COO^−^ group of D318 whilst a hydroxyl group on carbon-4′ makes a hydrogen bond with the amide oxygen of the N100 sidechain. Further interactions appear to exist between the hydroxyl groups on carbons 6′ and 3′ of the glucopyranose moiety and the sidechains of S59 and S60 (Fig. 2C). The main chain nitrogen of V61 may also make an interaction with the hydroxyl group on carbon 4′. In contrast, with ginsenoside Rd it is the disaccharide moiety attached to carbon-3 which is predicted to interact with the hP2X7 central vestibule glucose subsite, suggesting an inverted mode of binding compared to CK. None of the docking results for Rd placed the single glucopyranose moiety attached to carbon-20 within the putative binding site. Additional interactions were observed with docking of Rd to hP2X7, particularly stacking of the hydrophobic α-face of a glucose moiety onto the side chain of L320 (Fig. 1). Hydrophobic residues within the β-sheets likely enhance the interaction with the steroid backbone of the ginsenosides. In general, due to its smaller size CK may fit deeper within the cleft and this may explain its superior activity at hP2X7 compared to Rd. Furthermore, CK is more rigid than Rd and thus CK binding may involve a smaller entropic penalty.

Selected amino acid residues in these identified regions of the putative binding site on hP2X7 were then mutated using site-directed mutagenesis (S59, S60, N100, S101, D318, and L320) and the mutant hP2X7 receptors were stably expressed in HEK-293 cells. The G99EE mutant was generated to mimic the sequence found in human P2X4 receptors (QEENS) vs human P2X7 (QGNS) (Fig. 2D) since P2X4 can also be potentiated by ginsenosides^25^. Flow cytometry analysis indicated all the mutant hP2X7 receptors were expressed at the cell surface, although at lower levels than wild-type hP2X7 (Fig. S2).

### Loss of ginsenoside-induced potentiation of hP2X7 carrying D318 or L320 central vestibule mutations

**Figure 3:**
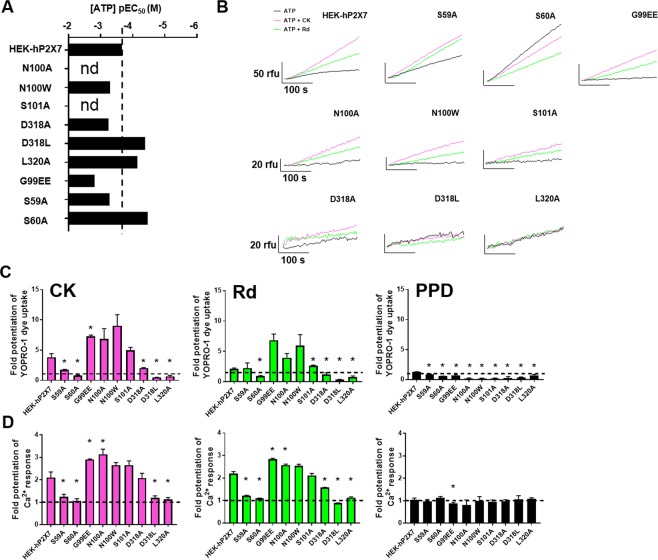
Mutations within the predicted ginsenoside binding site abolish potentiation of P2X7-dependent dye uptake and sustained Ca^2+^ responses. (**A**) ATP-induced dye uptake into HEK-P2X7 cells was measured at 37 °C using a fluorescent plate reader (Flexstation 3). YOPRO-1 (2 µM) was the membrane impermeant dye, all compounds were prepared in DMSO and experiments were performed in low divalent cation buffer. Representative dye uptake fluorescence traces for hP2X7 mutants vs WT following stimulation with 200 µM ATP plus DMSO (black), ATP plus 10 µM CK (pink) or ATP plus 10 µM Rd (green). (**B**) pEC_50_ values were determined for WT P2X7 and mutants following stimulation with a range of ATP concentrations (10 µM to 2 mM) using YOPRO-1 responses measured as area under curve (50–300 seconds). (**C**) Fold-potentiation was calculated with respect to the ATP control response using AUC data from 3 independent experiments. (**D**) ATP-induced intracellular Ca^2+^ responses were measured at 37 °C using Fura-2AM loaded cells. Fold-potentiation of the sustained phase of the ATP-induced calcium response was calculated by taking mean Fura-2 ratio between 150–300 seconds. The ginsenoside aglycone PPD was included as a negative control. Error bars represent SEM, *P < 0.05.

Activation of hP2X7 results in the formation of a large reversible secondary pore which allows cell impermeant large cationic molecules (such as the fluorescent dye YOPRO-1) to enter the cells, bind to DNA and fluoresce^5^. Stimulation with ATP resulted in uptake of YOPRO-1 by all hP2X7 mutants. N100A, N100W, S101A and G99EE mutations in hP2X7 resulted in poorer dye uptake responses to ATP (Fig. 3B). Conversely, the S60A, D318L, and D320A mutations in hP2X7 resulted in YOPRO-1 responses with increased sensitivity to ATP (Fig. 3A,B). Following the simultaneous addition of ATP plus CK, or ATP plus Rd, 3.82 ± 1.02 and 2.14 ± 0.21 fold increases respectively in WT hP2X7-dependent YOPRO-1 uptake were observed (Fig. 3C). Calculating fold-potentiation elicited by CK and Rd on the ATP responses demonstrated that ginsenoside potentiation of N100A, N100W, S101A, and G99EE mutations was enhanced compared to WT hP2X7, although this only reached statistical significance for the G99EE mutation (Fig. 3C). Notably, S60A, D318L, L320A mutations resulted in a complete loss of CK or Rd potentiation of ATP-dependent dye uptake responses (Fig. 3B,C). S59A and D318A mutants showed reduced potentiation by CK or Rd. The control ginsenoside, the aglycone protopanaxadiol (PPD) did not result in potentiation of ATP responses at WT hP2X7 or any of the hP2X7 mutants (Fig. 3C). We confirmed that ginsenoside potentiation was abolished for the D318L, L320A, S60A mutants when BzATP (30 µM) was used as the agonist and observed the same general trend as with ATP-induced dye uptake responses (Fig. S3A–C). Furthermore, ATP and BzATP-induced dye uptake responses at WT, S60A, D318L, and L320A hP2X7 could be inhibited by the selective P2X7 antagonist AZ10606120 (Fig. S4) and responses induced by agonist plus PAM could also be inhibited by AZ10606120. The exception to this was D318L mutant where BzATP + CK responses could not be inhibited by AZ10606120 (Fig. S4).

Due to the low dye uptake responses to agonists in several hP2X7 mutants we next tested ginsenoside potentiation of intracellular Ca^2+^ responses using fura-2AM loaded cells. Stimulation with either ATP or BzATP resulted in a transient peak response followed by a sustained increase in intracellular calcium (Figs S5A,B and S6A–C) as previously demonstrated^13^. CK and Rd induced a 2.10 ± 0.70 and 2.19 ± 0.16 fold increase in ATP-dependent sustained Ca^2+^ response in HEK-hP2X7, respectively. In agreement with the dye uptake data, effects of ginsenosides were amplified for the G99EE, N100A, N100W, and S101A mutations (Fig. 3D). S60A, D318L, and L320A mutations were again observed to be more sensitive to lower concentrations of ATP (Fig. S5A,B) and potentiation of ATP-induced intracellular Ca^2+^ responses by CK and Rd was dramatically reduced/abolished in S59A, S60A, D318L, and L320A mutants (Fig. 3D). To rule out the possible saturation of the fura-2 dye in S59A and L320A mutant responses, we repeated the experiments using a lower concentration of ATP (50 µM) and saw a similar effect; greatly reduced potentiation of ATP responses by CK (Fig. S7).

### Mutations S60A, D318L and L320A affect P2X7 sensitivity to agonist

**Figure 4:**
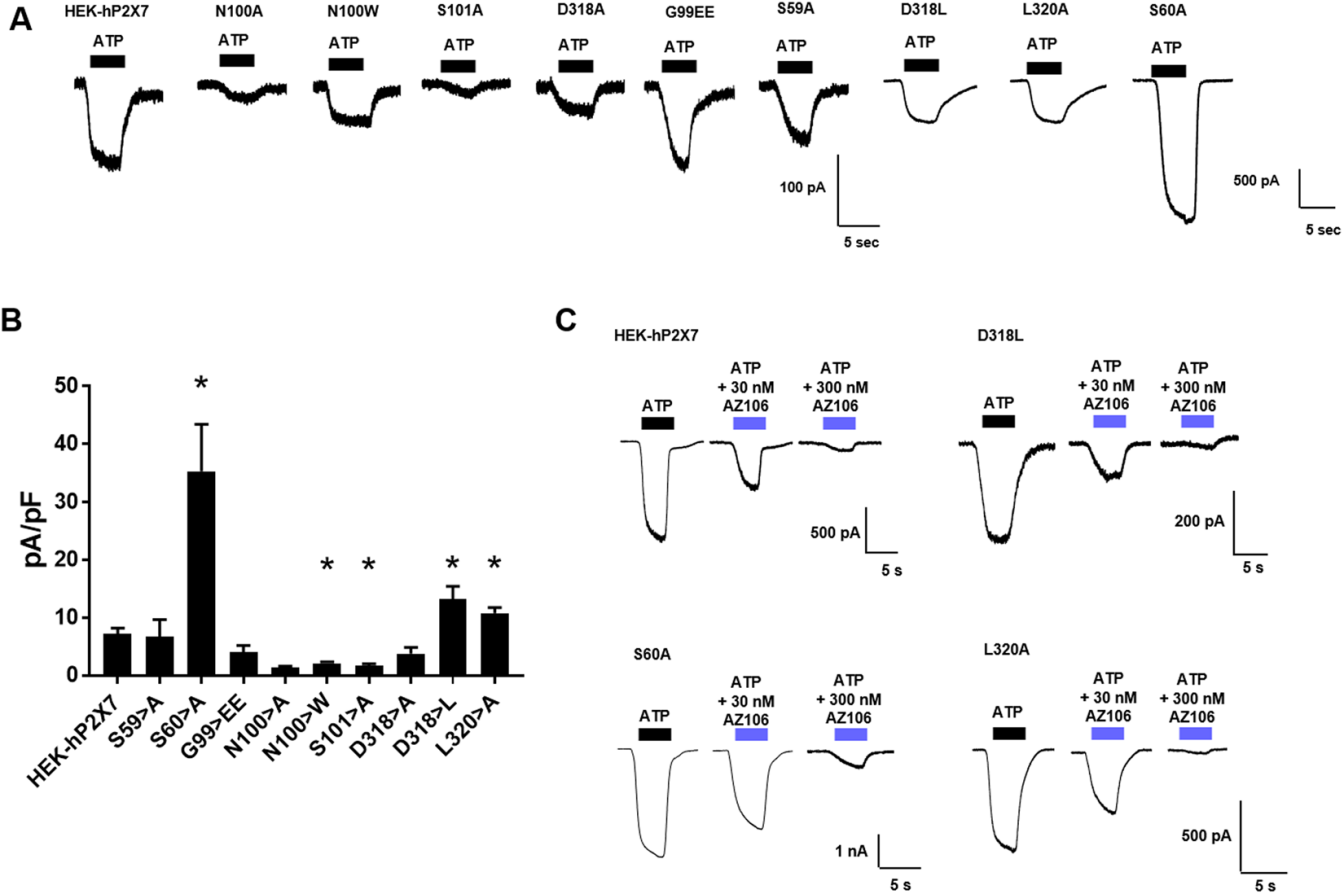
Patch clamp analysis reveals altered agonist responses in D318L, L320A and S60A mutants. Whole cell patch clamp recordings were performed at room temperature. Cells were voltage-clamped at −60 mV and responses to 5 second application of ATP (100 µM) measured from HEK-293 cells stably expressing WT or mutant hP2X7. **(A)** Representative traces are shown for ATP responses **(B)** Summary of normalised current amplitudes expressed as pA/pF (n = 5–25 cells). **(C)** Inhibition by AZ10606120 (30 nM, 300 nM) was tested in cells expressing D318L, S60A and L320A hP2X7 mutants and compared to WT hP2X7. An initial response to ATP was measured and then AZ10606120 applied to the cell for 1 minute followed by ATP in the presence of AZ10606120. Traces are representative of 3–5 cells.

Unexpectedly we found that mutating key residues predicted to interact with ginsenosides generated P2X7 receptors that were better able to respond to ATP or BzATP (Figs 3A–D and S3A–C). This was confirmed using whole cell patch clamp analysis of ATP responses and peak amplitudes were up to 5-fold larger in the S60A mutant (Fig. 4A). Responses to ATP (100 µM) could be completely inhibited by 300 nM AZ10606120 (Fig. 4C) in WT, D318L and L320A, however, S60A responses appeared to be somewhat less sensitive to this antagonist (Fig. 4C).

**Figure 5:**
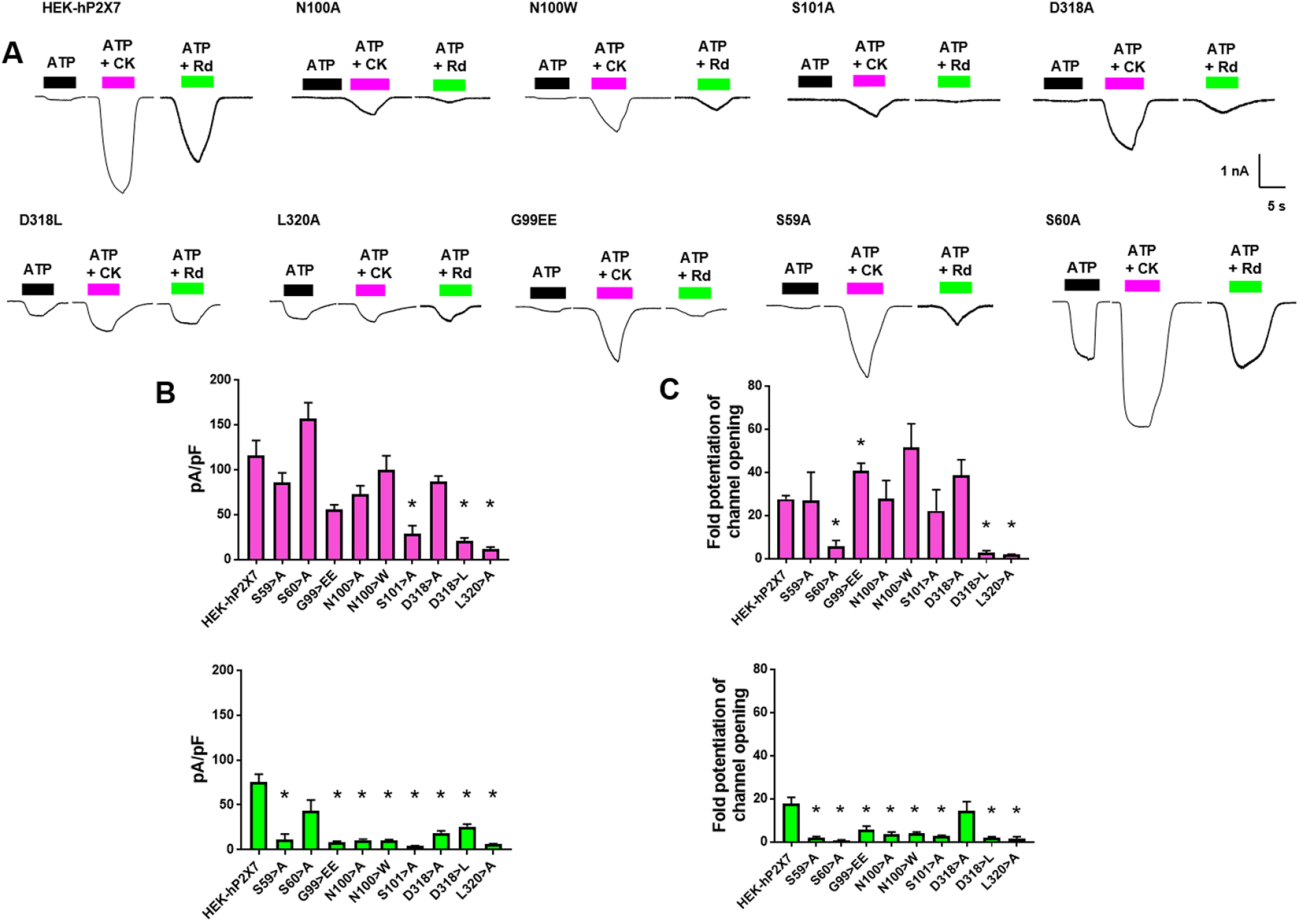
Electrophysiology reveals differences between CK and Rd potentiation of P2X7. (**A**) HEK-hP2X7 and central vestibule mutants were voltage clamped at −60 mV and either ATP alone (100 µM), ATP + CK (10 µM), or ATP + Rd (10 µM) was rapidly applied for 5 seconds prior to wash off. Traces are representative of 5–9 cells. (**B**) Summary of normalised current amplitudes expressed as pA/pF (n = 5–9 cells). (**C**) Potentiation of ATP responses by CK and Rd expressed as fold potentiation (normalised to ATP alone).

Patch clamp recordings revealed a much larger potentiation of ATP-induced inward currents by ginsenosides at WT hP2X7 than was estimated using the dye uptake and calcium measurements. CK and Rd potentiated ATP responses in WT hP2X7 cells by 27.61 ± 4.07 fold and 17.82 ± 6.51 fold respectively (Fig. 5A–C). Fold potentiation of ATP responses (100 µM) by CK and Rd was significantly reduced in S60A, D318L, and L320A mutants (Fig. 5A–C) as were BzATP-induced responses (Fig. S8). We also found that patch clamp recordings could better distinguish between CK-mediated and Rd-mediated potentiation of ATP-induced responses; G99EE, N100A, N100W, and S101A hP2X7 mutants demonstrated enhanced potentiation to CK but reduced potentiation by Rd (Fig. 5A–C). This may be due to the rapid delivery of agonist plus PAM to the cell and the inability of Rd to bind optimally during this timeframe.

### Ginsenoside binding enhances P2X7-mediated cell death

**Figure 6:**
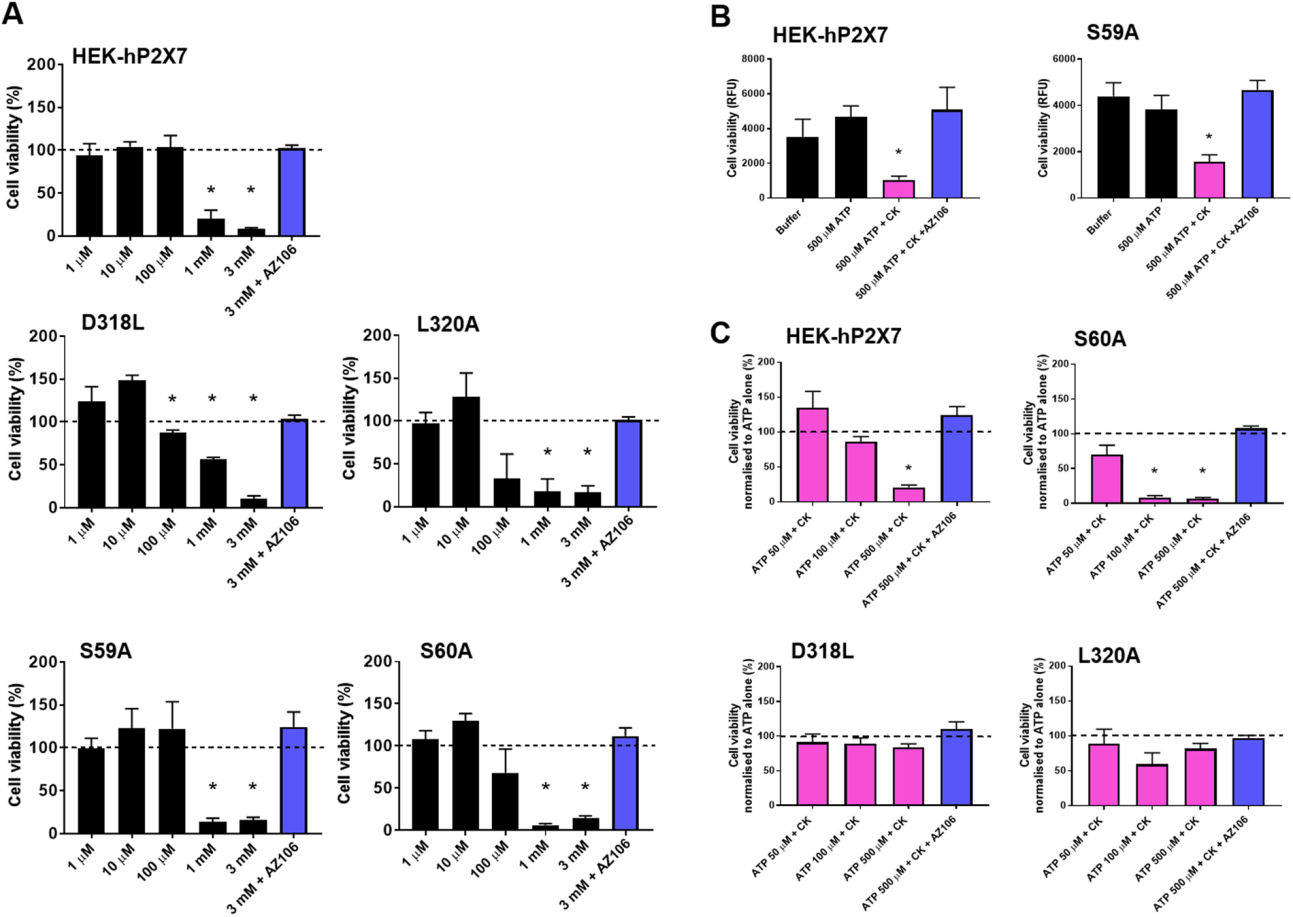
D318L and L320A mutations abolish the potentiating effect of CK on ATP-induced cell death. Cells were seeded at 5 × 10^3^ in a 96-well plate 24 h prior to stimulation with a range of ATP concentrations (1 µM–3 mM) or 3 mM ATP + AZ10606120 (10 µM) for a further 24 h. AlamarBlue was added for 2 h prior to measurement of fluorescence (RFU) using a Flexstation 3 plate reader. **(A)** Data is presented as the percentage mean cell viability compared to control media. **(B)** Cell viability was determined in the presence of 500 μM ATP + CK compared to 500 μM ATP alone for WT hP2X7 and S59A hP2X7. **(C)** Cell viability was determined in the presence of 50 μM ATP + CK, 100 μM ATP + CK, and 500 μM ATP + CK compared to each concentration of ATP alone for WT hP2X7, S60A, D318L and L320A hP2X7 mutants.

P2X7 is well known to be involved in the regulation of cell death^26^ and our previous work has demonstrated that CK enhances P2X7-dependent cell death in J774 murine macrophages^13^. We first determined whether S60A, D318L, and L320A mutants were more susceptible to ATP-induced cell death using an AlamarBlue cell viability assay (Fig. 6A). Cells expressing WT hP2X7 or S59A were killed by ATP concentrations >1 mM whereas S60A, D318L, and L320A mutants started to show reduced cell viability at ATP concentrations as low as 100 μM (Fig. 6A). AZ10606120 could inhibit cell death induced by 3 mM ATP in all cases (Fig. 6A–C). We have demonstrated that ginsenoside CK can induce cell death in combination with a non-lethal concentration of ATP (500 µM) in HEK-hP2X7 cells^25^. Here we show that CK in combination with ATP could reduce the viability of cells expressing WT hP2X7 or S59A-P2X7 and this was reversed by incubation with AZ10606120 (Fig. 6B). CK could still enhance cell death in the S60A mutant to ATP concentrations as low as 100 μM (Fig. 6C) likely due to retention of some potentiating action of CK at this mutant (5.88 ± 6.36 fold, Fig. 5). However, we found that CK could not enhance cell death in D318L or L320A P2X7 mutant cells (Fig. 6C) to various concentrations (50 μM, 100 μM, 500 μM) of ATP demonstrating that eliminating critical interacting residues on hP2X7 abolished all of the PAM effects of ginsenoside CK.

## Discussion

The main finding of this study is the identification of a putative binding site for ginsenosides located within the central vestibular region of P2X7 (Movie S1). This is the first PAM binding site to be described on the P2X7 receptor and furthermore, it is the first binding site to be identified within the central cavity (vestibule) of the P2X channel. Through targeted site-directed mutagenesis we have demonstrated that mutation of specific amino residues, namely the D318L, and L320A mutations within the lower body β-sheet region resulted in abolishment of CK and Rd effects on P2X7-dependent pore formation. Moreover, the D318L and L320A mutations resulted in abrogated Ca^2+^ influx and ATP-induced P2X7 channel opening. Furthermore, mutating the D318 and L320 residues was cytoprotective against the combined effects of ATP and CK at concentrations observed to kill HEK-hP2X7 cells.

Since the crystal structure of zfP2X4 in the apo and ATP bound states were published^19,23^, many studies have used homology models to predict the three-dimensional structure of other P2X receptors. The region we have investigated is the central vestibule which is accessed through the lateral portals, the proposed ion conduction pathway for P2X channels^27^. The β-sheet structures β-2 and β-14 from adjacent subunits in the trimeric P2X structure line the surface edge of these portals and additionally connect the ATP binding site to the transmembrane domains^28^. Upon ATP binding, rotational movements are thought to occur closing the ‘jaw’ around the agonist and allowing outward movement (flexing) of the edges of the lateral portals, widening the central vestibule^19^. We propose that upon opening of the P2X7 channel, a PAM binding site is revealed within the central vestibule and co-ordination of a chemical containing a glucopyranose moiety culminates in potentiation of the agonist-triggered response. Binding of the ginsenoside chemicals may stabilise the open state or may provide an enhanced movement of the connecting rods (β-sheets linking the ATP binding to TM movement). Roberts *et al*. demonstrated that locking of the β-sheets across the lateral portal by forcing a disulphide bridge between 60 C and 320 C in hP2X1 channels, inhibited channel responses to ATP^29^. Breaking this forced bridge using DTT effectively reversed the lock^29^. In addition, Samways *et al*. have demonstrated that certain residues in the central vestibule β-sheet region of hP2X4 were amenable to modification with MTSET^+^ ^27^. S59 and S62 (hP2X4 numbering) were mutated to cysteine residues and application of MTSET^+^ caused potentiation of ATP-responses at these mutant P2X4 channels. This suggests that residues in the lower body β2 sheet are accessible via lateral portals and that modifications can positively affect channel gating^27^.

There are few well described positive allosteric modulators of P2X channels. Within the family, ivermectin is best characterised for its actions on P2X4 and there is some evidence for PAM action in the region of the lateral portals. The binding site for ivermectin is thought to be much lower, residing within the transmembrane helices^30^. W46 and W50 in TM1 and D331 and M336 (rat P2X4 numbering) in TM2 are thought to contribute to the ivermectin binding site^31^ although residues E56 and D58 (hP2X4 numbering) may also contribute to the allosteric binding site for ivermectin^27^. Ivermectin is also thought to potentiate human P2X7 receptors^32^ but there is no evidence as yet whether this is due to interaction with a similar transmembrane binding site to P2X4. The possibility exists that ivermectin may bind to a site overlapping the ginsenoside site in the central vestibule however the following pieces of data may argue against this; firstly, ivermectin could not potentiate YOPRO-1 responses^32^ whereas ginsenosides can effectively potentiate YOPRO-1 responses (Fig. 3) and secondly, the potentiating effects of ivermectin were much smaller than the ginsenosides. Ivermectin is thought to have an effect on calcium permeability through the P2X4 channel (reducing the fractional calcium current, *Pf%*) and removal of the charged residue E51 at the top of TM1 abolished this effect^33^. Whilst we have not measured *Pf%* through P2X7 in this study, it is unlikely that ginsenosides affect calcium permeability due to the predicted binding site sitting well away from the transmembrane regions. We have measured sustained calcium levels in P2X7 expressing HEK cells and show that CK and Rd can significantly increase intracellular calcium levels (Fig. 4). Effects on membrane currents in cells clamped at −60 mV were also significant (Fig. 6) suggesting ginsenosides may increase cation conductance. This will potentially translate to changes in membrane potential which could be important in myeloid cells where ion fluxes (K^+^, Ca^2+^) may contribute to the activation of the NLRP3 inflammasome^34^.

Our finding that S60A, D318L, and L320A mutations created hP2X7 receptors with larger responses and higher sensitivity to ATP was somewhat unexpected. The overlap with these residues being highlighted as critical interacting residues with ginsenosides CK and Rd is unlikely to be coincidence and is suggestive that these residues make a difference to the ease of opening of the channel in response to agonist binding. Patch clamp data suggests the kinetics of channel opening are similar and there is no apparent difference in deactivation kinetics (Fig. 4A). Indeed, the already enhanced P2X7 activity in S60A mutant may underlie the reduction in ginsenoside-induced potentiation in this mutant. There is likely an upper limit to the movement of the β-sheets connecting the agonist binding site to the transmembrane domains. Furthermore, the altered sensitivity to AZ10606120 may reflect the amount of antagonist required to reverse the opening of this mutant channel.

Measuring the effects of the ginsenosides using whole cell patch clamp recordings revealed larger levels of potentiation of ATP responses than were shown in the plate reader fluorescence experiments (YOPRO-1, Ca^2+^). Additionally, these experiments exposed a difference between the potentiating effects of CK and Rd on the mutants. Partly this may be due to the co-exposure of the channel to agonist (ATP) plus ginsenoside for only 5 seconds before the agents were washed away. All of the mutants were poorly potentiated by Rd in comparison to CK (Fig. 6). This may be due to the strength and speed of binding of the modulators to the predicted central vestibule site. Ginsenoside CK being smaller than Rd and making more predicted interactions with lower body β-sheets, may bind better to the open state of the human P2X7 channel.

Our data on modulation of P2X7-induced cell death demonstrates that it is possible to enhance cell death with a positive allosteric modulator of P2X7. These experiments were performed over 24 hours, much longer than the immediate effects of channel activation and therefore show that modulation of channel activity can be translated into downstream outcomes. We confirmed that certain mutations (S59A, S60A, L320A) enhanced the sensitivity of P2X7 to ATP by showing reduced cell viability at low ATP concentrations (Fig. 6A). The S60A mutation did not completely abolish ginsenoside potentiation in the patch clamp experiments (Fig. 5A) and similarly, we still observed a potentiation in ATP-induced cell death in these cells. Importantly we observed that the D318L and L320A mutants were resistant to potentiation by CK confirming the lack of modulation of P2X7 channel activity in fluorescence/electrophysiology experiments.

In conclusion, the identification of a modulator binding site on P2X7 opens up new potential therapeutic benefits for disorders linked to dysfunctional P2X7 responses such as the defective killing of intracellular pathogens associated with loss-of-function polymorphisms in P2X7^35–37^. Furthermore, potentiators acting at this site might also be useful in inducing apoptotic cell death in tumour cells. It is also possible that we may be able to design antagonists to bind to this novel binding site potentially generating a series of open channel blockers to increase drug discovery opportunities for this important P2X receptor.

## Materials and Methods

### Cell culture

HEK-293 cells stably transfected with wild-type or mutant human P2X7 (hP2X7) receptors (EE-tagged hP2X7 in pcDNA3.1 plasmid) were maintained in DMEM/F-12 media containing L-glutamine (Life Technologies, Fisher Scientific) supplemented with 10% FBS (South American origin, Gibco), 10000 U mL^−1^ penicillin and 10 mg mL^−1^ streptomycin (Life Technologies, Fisher Scientific) with selection under 400 μg mL^−1^ G418 (Life Technologies, Fisher Scientific). All cells were maintained at 37 °C in a 5% CO_2_ humidified incubator.

### Mutagenesis

Point mutations were introduced into the WT hP2X7 plasmid using the Stratagene Quikchange II site-directed mutagenesis kit (Agilent Technologies). Primers were purchased from Sigma Aldrich. PCR was performed for 16–18 cycles using Pfu turbo polymerase and products digested with DpnI for 1 hour at 37 °C. NEB 5-alpha F’*I*^*q*^ competent E.coli high efficiency cells (New England Biolabs, UK) were transformed with 5 µl of digested product and colonies selected following growth at 37 °C for 16 hours. Plasmids were extracted using Qiagen miniprep kit and mutations verified by sequencing (Eurofins Genomics).

### Flow cytometry

To quantify cell surface expression of hP2X7, 1 × 10^5^ cells were pelleted prior to resuspension in primary mouse anti-hP2X7 antibody (clone L4, a kind gift from Associate Professor Ronald Sluyter, University of Wollongong, Australia) at a dilution of 1:100 in PBS/0.5% BSA buffer. Cells were incubated for 1 hour on ice. Following a washing step, cells were resuspended in secondary goat anti-mouse IgG AlexaFluor 488 (Fisher Scientific) at a dilution of 1:100 and incubated in the absence of light for 1 h on ice. Cells were washed and fluorescence was measured on a CytoFLEX flow cytometer (Beckman Coulter; laser excitation, 488 nm; emission detection, 533/30 nm). Data was analysed using CytExpert software (Beckman Coulter; version 2.1). All washes were conducted in PBS containing 0.5% (w/v) BSA at 300 *g* for 5 mins.

### Dye uptake experiments

Cells were plated at 2.5 × 10^4^ cells per well the day before experiments into poly-D-lysine (Millipore) coated 96-well plates (NUNC, Fisher Scientific). The membrane impermeant dye YOPRO-1 iodide (Life Technologies, Fisher Scientific) was used at a final concentration of 2 μM in low divalent buffer (145 mM NaCl, 5 mM KCl, 0.2 mM CaCl_2_, 13 mM glucose, 10 mM HEPES, pH 7.3, osm 300–310). Data was acquired on a Flexstation 3 plate reader (Molecular Devices) using excitation wavelength 490 nm, emission wavelength 520 nm and 6 reads per well. ATP ± ginsenosides at 10X final concentration was automatically injected at 40 seconds from a 96-well V-bottomed drug plates (Greiner). Pipette height was set to 180 µl and the rate of injection was 4. Data was analysed as AUC between 50 and 300 seconds using SoftMax Pro v5.4 software (Molecular Devices).

### Calcium measurements

Cells were plated at 2.5 × 10^4^ cells per well the day before experiments into poly-D-lysine coated 96-well plates. Cells were loaded with 2 μM Fura-2-acetoxymethyl (AM) calcium indicator dye (Hello Bio, UK) in standard HBSS buffer containing 250 µM sulfinpyrazone for 40 minutes at 37 °C. This loading buffer solution was removed and replaced with standard low divalent buffer containing 250 µM sulfinpyrazone. A Flexstation 3 plate reader was used to acquire data using dual excitation wavelengths 340 nm and 380 nm with an emission wavelength of 520 nm, 3 reads per well. ATP ± ginsenosides/vehicle at 10X final concentration was injected after 40 seconds. Data was analysed as AUC between 150 seconds and 300 seconds using SoftMax Pro software (Molecular Devices).

### Patch-clamp electrophysiology

Stably expressing HEK-hP2X7 cells were plated onto 13 mm glass coverslips 24 hours prior to use. Membrane currents were recorded in the whole-cell patch-clamp configuration using an EPC10 amplifier (HEKA Elektronik) and borosilicate glass electrodes (TF-150 World Precision Instruments), resistance 3–8 Ω when filled with standard internal solution (145 mM NaCl, 10 mM HEPES, 10 mM EGTA, pH 7.3). Cells were voltage clamped at −60 mV and dialysis of intracellular contents was performed for 1 minute prior to experimental procedures. Cells were continuously perfused by gravity feed with standard divalent buffer solution (145 mM NaCl, 5 mM KCl, 2 mM CaCl_2_, 1 mM MgCl_2_, 13 mM glucose, 10 mM HEPES, pH 7.3) prior to seal formation, and low divalent buffer solution was used for agonist and PAM application in most experiments. Agonists were applied using a computer-controlled fast-flow system (RSC-160; Bio-Logic Scientific Instruments) with the perfusion capillaries placed in close proximity to the cell under investigation.

### Cell viability assays

Cells were plated at 5 × 10^3^/well in Nunc Edge 96-well plates (Scientific Lab Supplies, UK) 24 hours prior to stimulation with increasing concentrations of ATP (1 μM–3 mM), or a combination of ATP and CK (500 μM and 10 μM, respectively) in the presence or absence of AZ10606120 (10 μM), for 24 h. Following incubation, resazurin (0.1 mg/ml in PBS, Sigma Aldrich) was added to cells for 2 h at 37 °C and fluorescence was measured on a Flexstation 3 plate reader (laser excitation, 570 nm; emission detection, 600 nm).

### P2X7 homology model

The coordinates of zfP2X4 in the ATP-bound open state (PDB: 4DW1) were used as a template. The sequence of human P2X7 was aligned to the template using the ClustalW algorithm^38^. The Schrödinger Prime software was used to construct an energy-based all-atom model with the OPLS3 forcefield, keeping the ATP coordinates from the template. Regions of low sequence homology were refined using extended serial loop sampling in the Prime software.

### Induced fit docking

Three-dimensional models of the ginsenosides CK and Rd were generated using the LigPrep software within the Schrödinger Maestro suite. The OPLS3 forcefield was used to generate 32 low-energy conformers for each ginsenoside. Induced fit docking was performed using the automated extended sampling protocol in the Schrödinger Maestro suite. This first performed several initial docking runs, in which sidechains were either trimmed, or their van der Waals potentials softened according to their flexibility. Side chains were then rebuilt and those within 5 Å of the ligand were optimised using Prime^39^. Ligands were then re-docked to the new receptor structure using the Glide SP algorithm^40^ and standard potentials. Structures within 30 kcal mol^−1^ of the lowest energy structure were retained.

The receptor grid was centred on the highest-scoring potential binding site, found using Sitemap^41^, and had a cubic box with dimensions of 30 Å. Each of the 32 conformers were docked using the extended sampling protocol. For each ginsenoside, the resulting poses were clustered by heavy atom RMSD using the average-linkage method, and a representative structure was chosen from the model closest to the centroid of the most populated cluster. For CK and Rd the most populated cluster made up 27% and 59% of all solutions respectively.

### Data analysis

All graphs were plotted and statistical tests performed using GraphPad Prism v7. Significance was taken as P < 0.05.

## Supplementary information

Supplementary Information

Movie S1

## Data Availability

The data generated during the current study are available from the corresponding author on reasonable request. Molecular models of P2X7 are available at https://bit.ly/2DhW2fF.
