# Supplementary material for: Mapping a novel positive allosteric modulator binding site in the central vestibule region of human P2X7
Source: Sci Rep. 2019 Mar 1;9:3231. doi: 10.1038/s41598-019-39771-5 (PMC6397193; doi:10.1038/s41598-019-39771-5)
Supplement: Supplementary file 1 — Supplementary Information [file 41598_2019_39771_MOESM1_ESM.pdf]

Supplementary Information

Mapping a novel positive allosteric modulator binding site in the central vestibule region of human P2X7

Stefan Bidula[1], Brett A. Cromer [2,3], Samuel Walpole[1], Jesus Angulo[1] & Leanne Stokes[1*]

[1]*School of Pharmacy, University of East Anglia, Norwich Research Park, Norwich,NR4 7TJ United Kingdom*

[2]*School of Medical Sciences, RMIT University, Bundoora, VIC 3083, Australia*

[3]*Department of Chemistry & Biotechnology, Swinburne University of Technology, Hawthorn, VIC, Australia (current address)*

*Corresponding author. l.stokes@uea.ac.uk

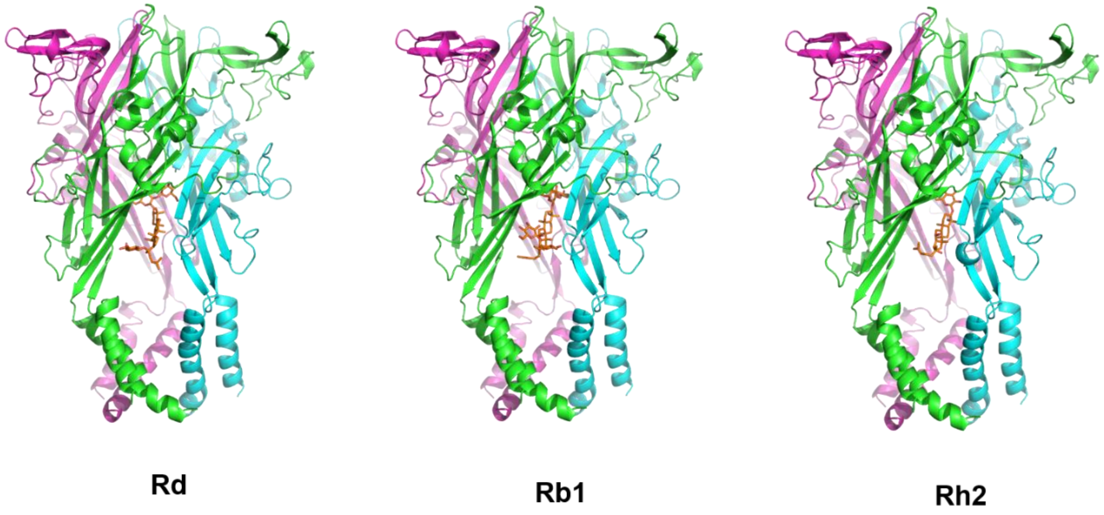

Fig. S1. Docking of Rd, Rb1, and Rh2 ginsenosides to central vestibule region of hP2X7. Representation of a homology model of hP2X7 trimer in the open state with ginsenosides Rd, Rb1 or Rh2 docked in the central vestibular region. Each subunit in the trimer is differentially coloured.

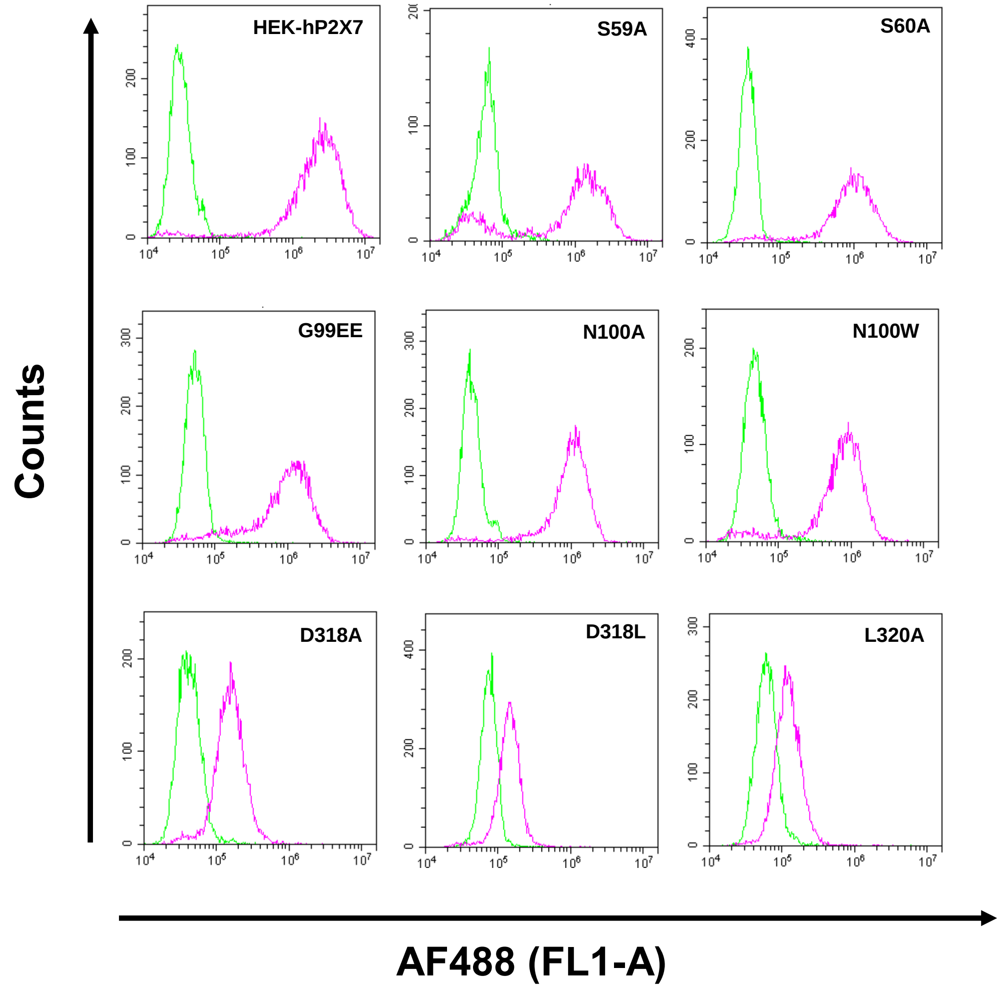

Fig. S2. All mutant hP2X7 receptors are expressed on the surface of transfected HEK293 cells. To quantify surface expression, 1 x 10^5 cells were stained with mouse anti-hP2X7 primary antibody (clone L4, 1:100 dilution), prior to staining with secondary goat anti-mouse IgG AlexaFluor 488. Fluorescence was measured on a CytoFLEX flow cytometer (excitation, 488 nm; emission 533/30 nm) and analysed using CytExpert software.

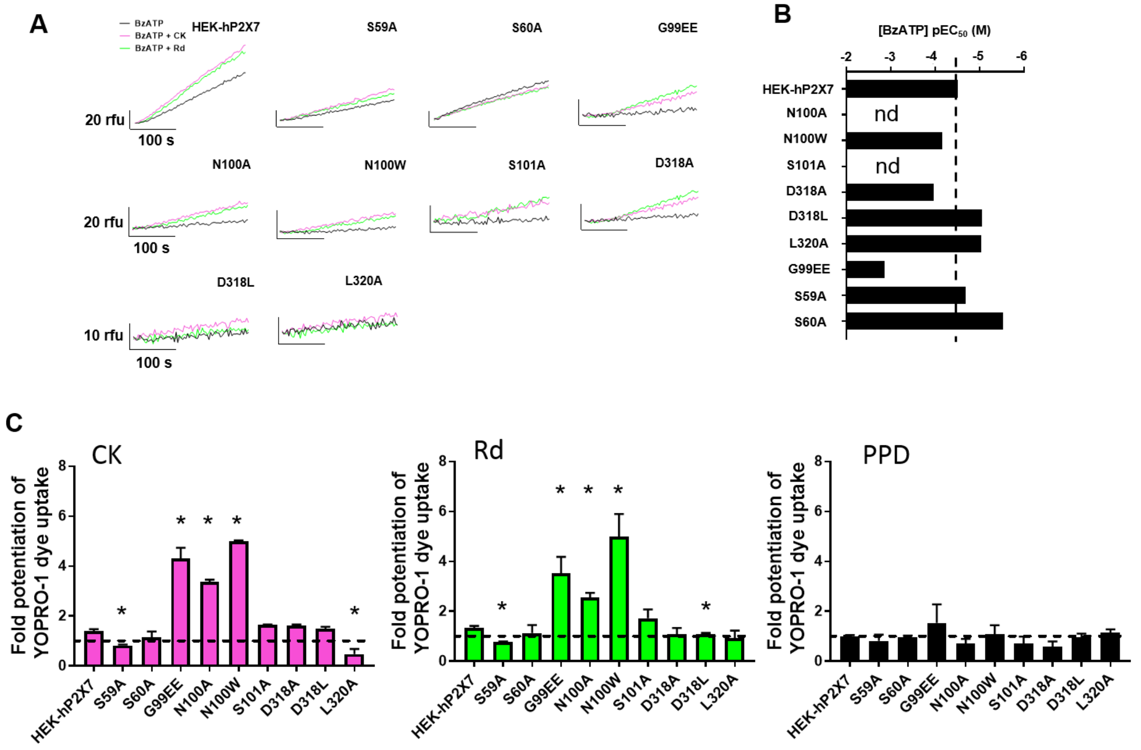

Fig. S3. Mutations within the predicted ginsenoside binding site abolish potentiation of BzATP-dependent dye uptake. BzATP-induced dye uptake into HEK-P2X7 cells was measured at 37°C using using a Flexstation 3. YOPRO-1 (2 µM) was the membrane impermeant dye, all compounds were prepared in DMSO and experiments were performed in low divalent cation buffer. (A) Representative dye uptake fluorescence traces for hP2X7 mutants vs WT following stimulation with 30 µM BzATP plus DMSO (black), BzATP plus 10 µM CK (pink) or BzATP plus 10 µM Rd (green). (B) pEC_{50} values were determined for WT P2X7 and mutants following stimulation with a range of BzATP concentrations (0.3 µM to 300 µM). YOPRO responses were measured as area under curve (50-300 seconds). (C) Fold-potentiation was calculated using AUC data from 3 independent experiments. The ginsenoside aglycone PPD was included as a negative control. Error bars represent SEM, * represent $P<0.05$ from one-way ANOVA with multiple comparisons post-test.

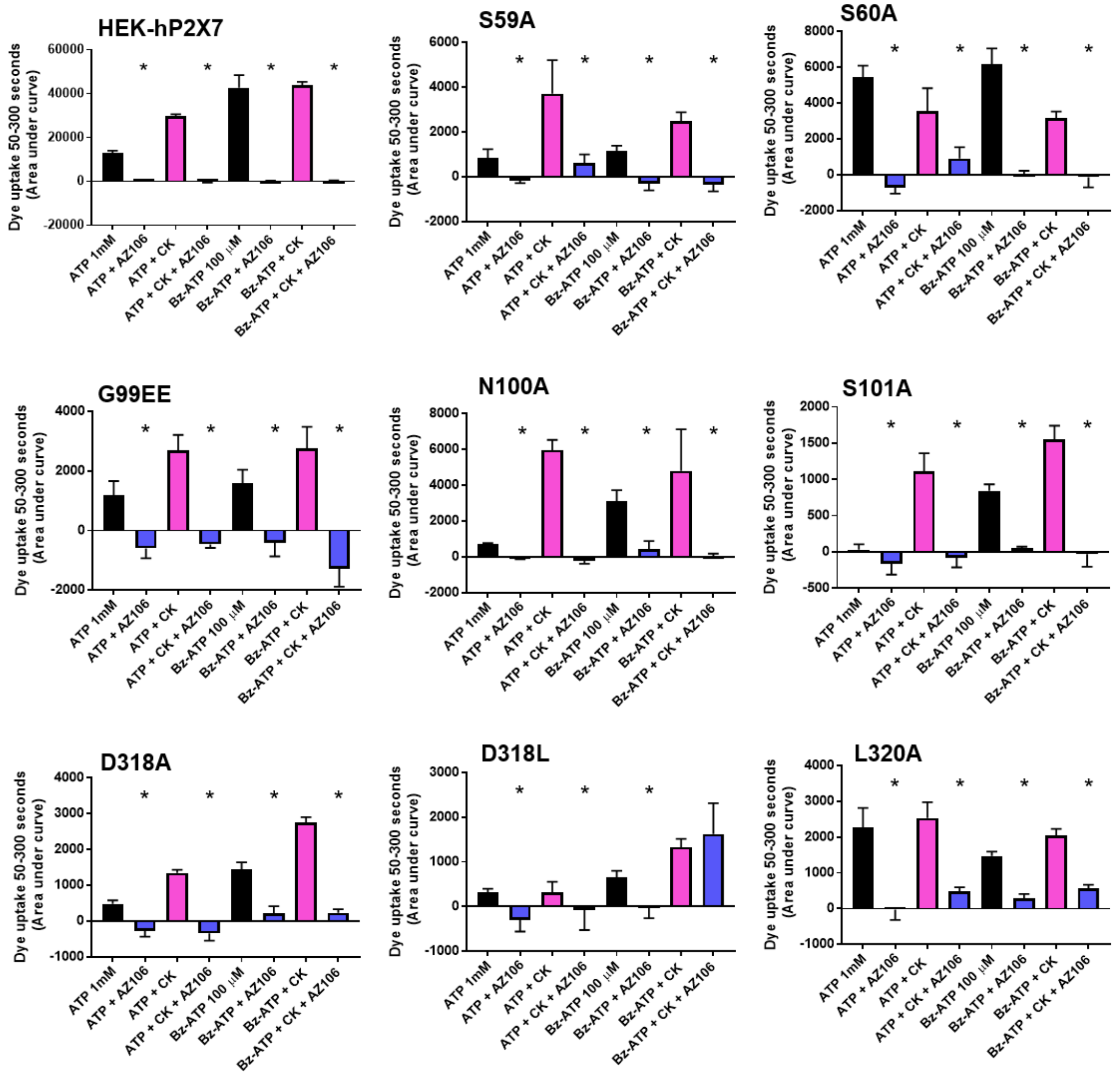

Fig. S4. Potentiation of P2X7-dependent pore formation by CK can be inhibited by using the selective hP2X7 antagonist AZ10601620. ATP- or BzATP-induced dye uptake was measured at 37°C using YOPRO-1 (2 µM) as the membrane impermeant dye. HEK-hP2X7 and central vestibular mutants were stimulated with ATP (1 mM) or BzATP alone (100 µM) ± CK (10 µM) and RFU were measured following excitation at 490 nm and emission recorded at 520 nm using a Flexstation 3 fluorescent plate reader. Responses were inhibited by pre-incubating cells with AZ10606120 (10 µM) for 10 min prior to stimulation. All compounds were prepared in DMSO and experiments were performed in low divalent cation buffer. Error bars are SEM, * represent $P<0.05$ by one-way ANOVA with multiple comparisons post-test.

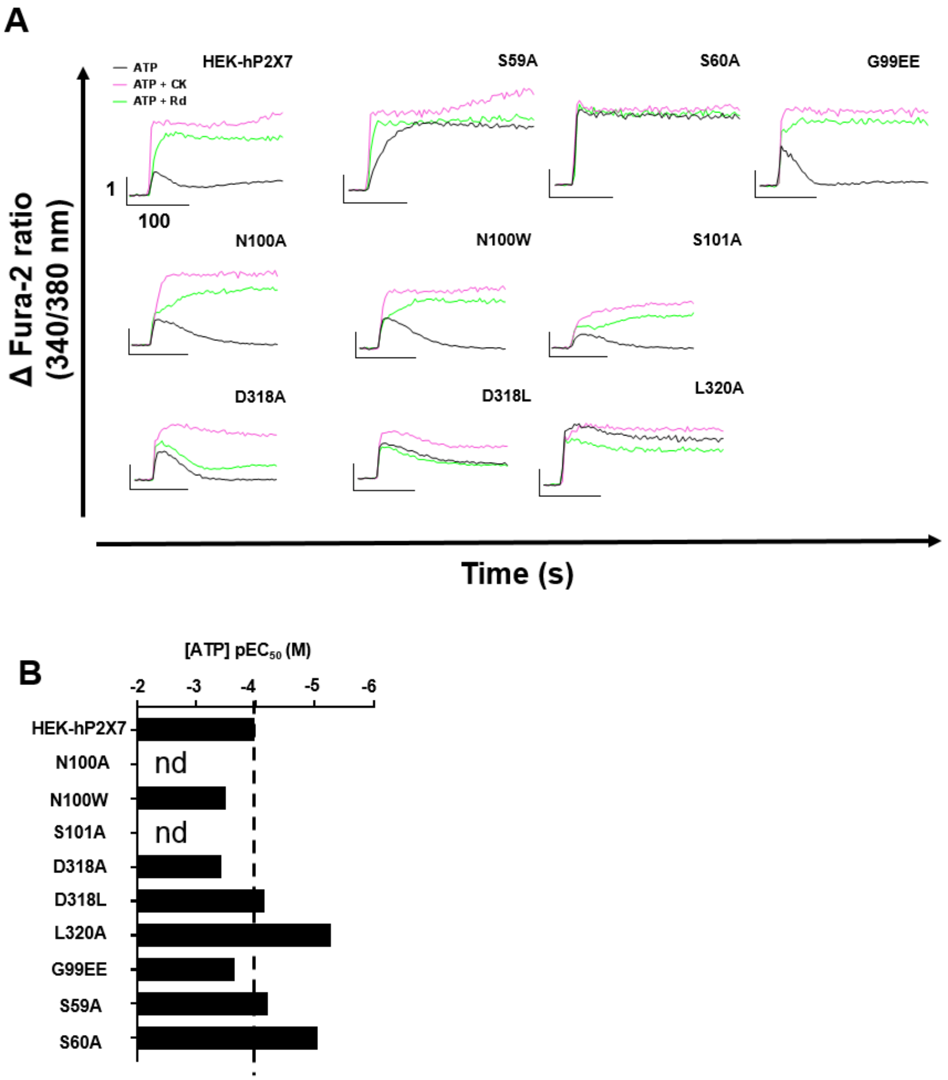

Fig. S5. Mutations within the predicted ginsenoside binding site can inhibit P2X7 sustained Ca^{2+} responses. ATP-induced intracellular Ca^{2+} responses were measured at 37°C using Fura-2. (A) Representative fluorescence traces for hP2X7 mutants showing stimulation with ATP (200 µM) with either CK (pink) or Rd (green) (both 10 µM). (B) pEC_{50} values were determined for WT P2X7 and mutants following stimulation with a range of ATP concentrations (10 µM to 2mM).

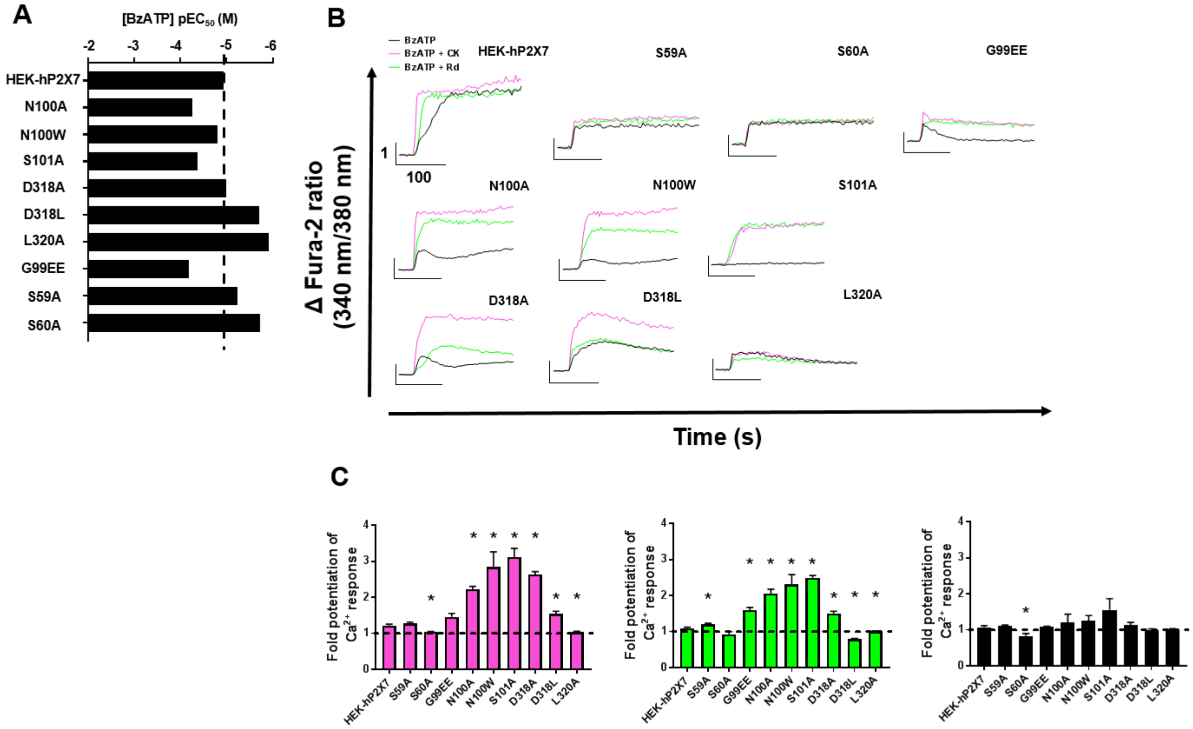

Fig. S6. Mutations within the predicted ginsenoside binding site can inhibit P2X7 sustained Ca^{2+} responses. BzATP-induced intracellular Ca^{2+} responses were measured at 37°C using Fura-2. (A) pEC_{50} values were determined for WT P2X7 and mutants following stimulation with a range of BzATP concentrations (0.3 µM to 300 µM). (B) Representative fluorescence traces for hP2X7 mutants showing stimulation with BzATP (30 µM) with either CK (pink) or Rd (green) (both 10 µM). (C) Fold-potentiation of the sustained phase of the BzATP-induced calcium response was calculated by taking mean Fura-2 ratio between 150-300 seconds. Error bars are SEM, * represent $P<0.05$ by one-way ANOVA with multiple comparisons test.

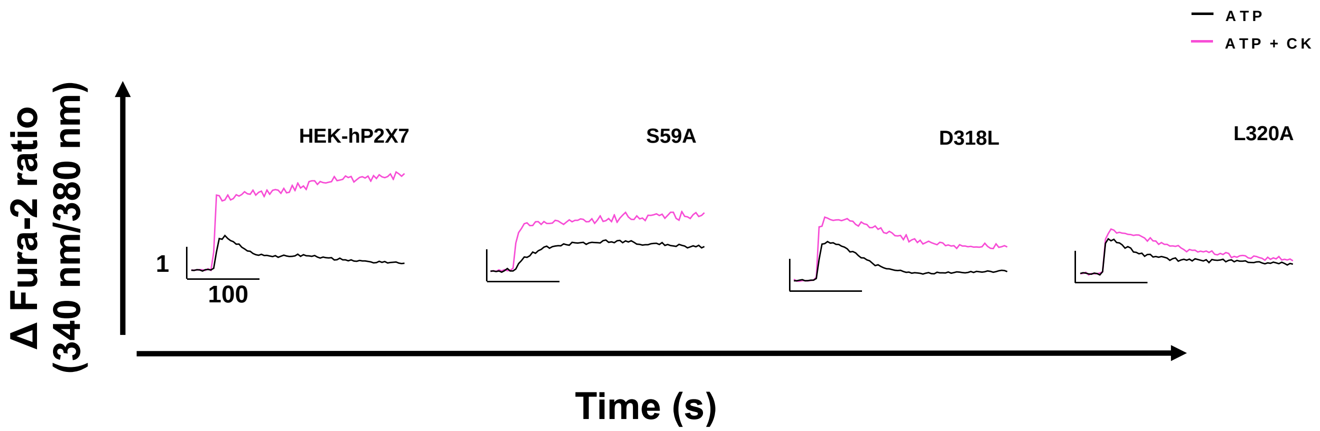

Fig. S7. Reduced potentiation of ATP-induced calcium responses in L320A and D318L P2X7 mutants. ATP-induced intracellular Ca^{2+} responses were measured at 37°C using Fura-2. Representative fluorescence traces for hP2X7 mutants showing stimulation with ATP (50 µM) with CK 10 µM (pink).

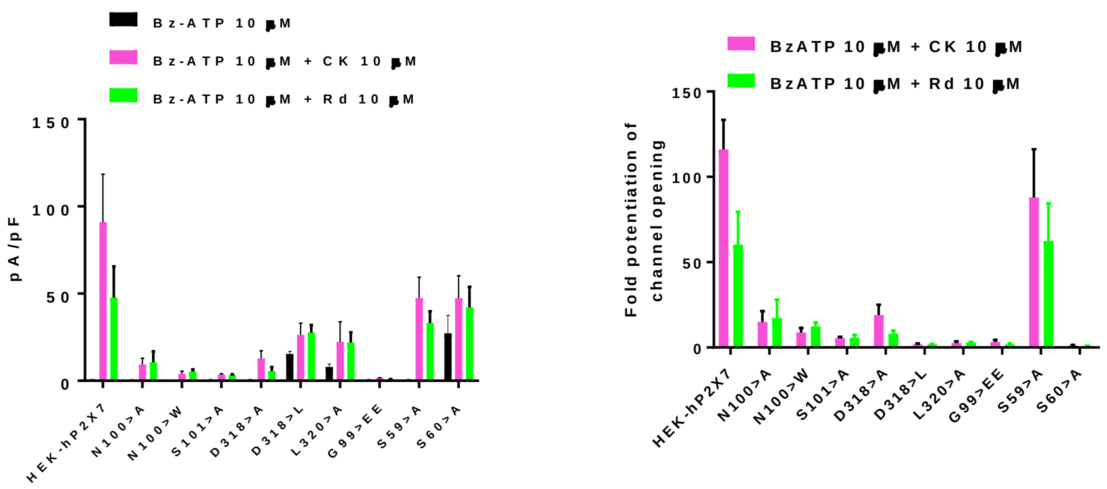

Fig. S8. Reduced potentiation of BzATP-induced inward currents in S60A, D318L and L320A P2X7 mutants. (A) HEK-hP2X7 and central vestibule mutants were voltage clamped at -60 mV and either BzATP alone (10 µM), BzATP + CK (10 µM), or BzATP + Rd (10 µM) was rapidly applied for 5 seconds prior to wash off. Summary of normalised current amplitudes expressed as pA/pF (n=5-9 cells). **(B)** Potentiation of BzATP responses by CK and Rd expressed as fold potentiation (normalised to BzATP alone).

Movie S1. Location of the central vestibule binding site in hP2X7 displaying ginsenoside CK bound to lower body β-sheets. Three molecules of ATP are shown as red spheres; ginsenoside CK is displayed as orange sticks. Movie was created in Chimera by Dr Marco M D Cominetti.
